# MatMRI and MatHIFU: software toolboxes for real-time monitoring and control of MR-guided HIFU

**DOI:** 10.1186/2050-5736-1-7

**Published:** 2013-06-04

**Authors:** Benjamin Zaporzan, Adam C Waspe, Thomas Looi, Charles Mougenot, Ari Partanen, Samuel Pichardo

**Affiliations:** 1 Thunder Bay Regional Research Institute, Thunder Bay, Ontario P7B 6V4, Canada; 2 Electrical Engineering, Lakehead University, Thunder Bay, Ontario P7B 5E1, Canada; 3 The Hospital for Sick Children, Toronto, Ontario M5G 1X8, Canada; 4 Philips Healthcare, Toronto, Ontario M3C 3E9, Canada; 5 Philips Healthcare, Cleveland, OH 44143, USA

## Abstract

**Background:**

The availability of open and versatile software tools is a key feature to facilitate pre-clinical research for magnetic resonance imaging (MRI) and magnetic resonance-guided high-intensity focused ultrasound (MR-HIFU) and expedite clinical translation of diagnostic and therapeutic medical applications.

In the present study, two customizable software tools that were developed at the Thunder Bay Regional Research Institute are presented for use with both MRI and MR-HIFU. Both tools operate in a MATLAB^®;^ environment. The first tool is named MatMRI and enables real-time, dynamic acquisition of MR images with a Philips MRI scanner. The second tool is named MatHIFU and enables the execution and dynamic modification of user-defined treatment protocols with the Philips Sonalleve MR-HIFU therapy system to perform ultrasound exposures in MR-HIFU therapy applications.

**Methods:**

MatMRI requires four basic steps: initiate communication, subscribe to MRI data, query for new images, and unsubscribe. MatMRI can also pause/resume the imaging and perform real-time updates of the location and orientation of images. MatHIFU requires four basic steps: initiate communication, prepare treatment protocol, and execute treatment protocol. MatHIFU can monitor the state of execution and, if required, modify the protocol in real time.

**Results:**

Four applications were developed to showcase the capabilities of MatMRI and MatHIFU to perform pre-clinical research. Firstly, MatMRI was integrated with an existing small animal MR-HIFU system (FUS Instruments, Toronto, Ontario, Canada) to provide real-time temperature measurements. Secondly, MatMRI was used to perform T2-based MR thermometry in the bone marrow. Thirdly, MatHIFU was used to automate acoustic hydrophone measurements on a per-element basis of the 256-element transducer of the Sonalleve system. Finally, MatMRI and MatHIFU were combined to produce and image a heating pattern that recreates the word ‘HIFU’ in a tissue-mimicking heating phantom.

**Conclusions:**

MatMRI and MatHIFU leverage existing MRI and MR-HIFU clinical platforms to facilitate pre-clinical research. MatMRI substantially simplifies the real-time acquisition and processing of MR data. MatHIFU facilitates the testing and characterization of new therapy applications using the Philips Sonalleve clinical MR-HIFU system. Under coordination with Philips Healthcare, both MatMRI and MatHIFU are intended to be freely available as open-source software packages to other research groups.

## Background

Pre-clinical research is a fundamental step in the development of new diagnostic and therapeutic applications. However, this work is often time-consuming and may require multi-disciplinary teams of physicists, medical doctors, biologists, and engineers. Magnetic resonance-guided high-intensity focused ultrasound (MR-HIFU) is a novel treatment modality that enables targeted thermal therapy under magnetic resonance imaging (MRI) guidance. In terms of capital cost, the access to an MRI scanner and to an MRI-compatible HIFU device represents one of the most expensive investments for a research team. It is therefore desirable that this investment can be leveraged to its maximum potential to conduct various pre-clinical MR-HIFU projects. The Sonalleve system (Philips Healthcare, Vantaa, Finland) is a clinical MR-HIFU platform used for thermal therapies and operates in tandem with 1.5- and 3.0-T Achieva and Ingenia (Philips Healthcare, Best, the Netherlands) MRI scanners. The MR-HIFU device communicates with the MRI scanner to obtain images that are used for real-time calculations of temperature maps using a technique based on the water-proton resonance frequency shift (PRFS) [1]. This MR-HIFU platform includes a transducer with 256 independent elements operating at a frequency between 1.0 and 1.5 MHz and has a focusing length of 12 cm and a diameter of 13 cm. The system also includes the driving electronics and a motorized subsystem that allows positioning the transducer with 5 degrees of freedom. The system is clinically approved for the treatment of uterine fibroids in several regions including Canada, Europe, India, and Korea [2].

**Figure 1:**
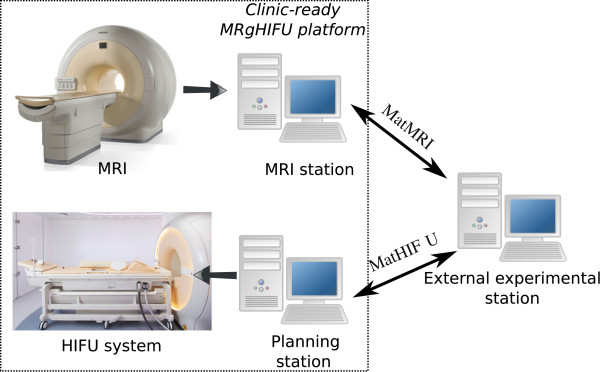
**Software toolboxes MatMRI and MatHIFU. **Using an external computer, MatMRI enables communication with an MRI scanner and MatHIFU allows controlling an MR-compatible HIFU system.

The availability of open and versatile software tools is essential to facilitate pre-clinical research and accelerate clinical translation of both diagnostic and therapeutic medical applications. Pre-clinical research for MRI and MR-HIFU often requires custom software modifications that may not be compatible with installations in the clinic. In the present study, two software tools (Figure 1) are presented for use with both MRI and MR-HIFU. These tools were developed at the Thunder Bay Regional Research Institute (TBRRI). The first software tool is named MatMRI, and it allows direct communication with a Philips MRI scanner in a MATLAB^®;^ environment (MathWorks, Natick, MA, USA). The choice of MATLAB^®;^ is due to the high popularity of this computational tool in many research laboratories. As shown in Figure 1, MatMRI is executed in an external computer and does not require any modification to the clinically approved MRI scanner software. MatMRI can be used to perform real-time dynamic acquisition of MR images that can be further processed, e.g., to calculate temperature changes. Analogous to MatMRI, the second software tool is named MatHIFU, and it is used to control the Philips Sonalleve clinical MR-HIFU system in a MATLAB^®;^ environment. MatHIFU allows executing user-defined treatment protocols that can be used in various MR-HIFU therapy applications, including thermal ablation ( >55°C), mild hyperthermia ( 40°C– 55°C), and HIFU-mediated drug delivery [3-7]. MatMRI and MatHIFU can be combined or used independently. Both tools were designed and developed to foster collaboration between research groups and are intended to be freely available for groups performing pre-clinical research using Philips MRI or MR-HIFU hardware.

## Results and discussion

### Results

To showcase results achieved through the use of MatMRI and MatHIFU, four applications that were developed at TBRRI and the Hospital for Sick Children (Toronto, Ontario, Canada) are presented. These applications show the ability of MatMRI and MatHIFU to control MR imaging in real time for pre-clinical animal studies, generate thermal maps using T2-based thermometry, drive individual transducer elements to perform acoustic characterization, and execute custom ablation patterns.

#### Integration of MatMRI with a small animal MR-HIFU system for the treatment of abscesses in a murine model

**Figure 2:**
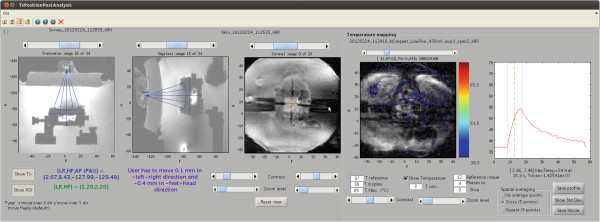
**Graphical user interface for the treatment of MRSA-related abscesses in a mouse model using MR-HIFU. **MatMRI was used to capture multi-slice 2D images intended for planning as well as dynamic T1-based, single-slice images for the thermometry. In this example, a treatment with an acoustic power of 25 W was used to produce an elevation of temperature from 37°C to 55°C at the center of an abscess.

MatMRI was integrated with existing software used to control a table designed for small animal MR-HIFU studies (FUS Instruments, Toronto, Ontario, Canada). Experiments were conducted to test the hypothesis that MR-HIFU can be used as a therapeutic option for the treatment of abscesses related to methicillin-resistant *Staphylococcus aureus* (MRSA) [8]. The animal protocol was approved by the Animal Care Committee of Lakehead University (AUP 08 2012). A 50- *μ*L subcutaneous injection of an MRSA strain, USA-400 bacteria, at a concentration of 7 × 10^3^/*μ*L was performed on the left flank of BALB/c mice, and an abscess of 6 ±2 mm in length formed after 48 h. The abscess was targeted using a transducer operating at 3 MHz with a focal length of 50 mm and diameter of 32 mm. The focal point was positioned 2 mm underneath the abscess, and an ultrasound exposure was applied over 9 s with an acoustic power of 25 or 35 W. Temperature maps were calculated from the coronal MR images of the subcutaneous region of the left flank using the PRFS technique. Magnetic drift was monitored in the non-heated muscle region, and a correction was applied. MR imaging parameters for thermometry were as follows: field of view (FOV) = 80 mm, pixel size = 1 mm, slice thickness = 3 mm, echo time/repetition time (TE/TR) = 16/23 ms, flip angle = 19°, acquisition matrix = 68×63, reconstruction matrix = 80, echo train length (ETL) = 9, number of excitations (NEX) = 1, and dynamic time = 0.35 s. Figure 2 shows a screenshot of the graphical user interface used to monitor and control the experiments.

End points were at days 1 and 4 after MR-HIFU treatment. For each end point, 18 animals were randomly assigned into three groups with 6 animals each: control, treatment with an acoustic power of 25 W, and treatment with an acoustic power of 35 W. Results indicated that an exposure with an acoustic power of 35 W was able to induce a significant ( *p* < 0.05) reduction of the bacteria concentration in the abscess when compared to non-treated abscesses and low-power exposures. The bacteria concentration (mean ± standard deviation) at the 4-day end point was 1.0 ± 1.3 × 10^4^, 0.6 ± 0.6 × 10^4^, and 0.09 ± 0.2 × 10^4^ colony-forming units/ *μ*L for the control, low power, and high power groups, respectively.

#### T2-based thermometry with MatMRI for the MR-HIFU treatment of the bone marrow

**Figure 3:**
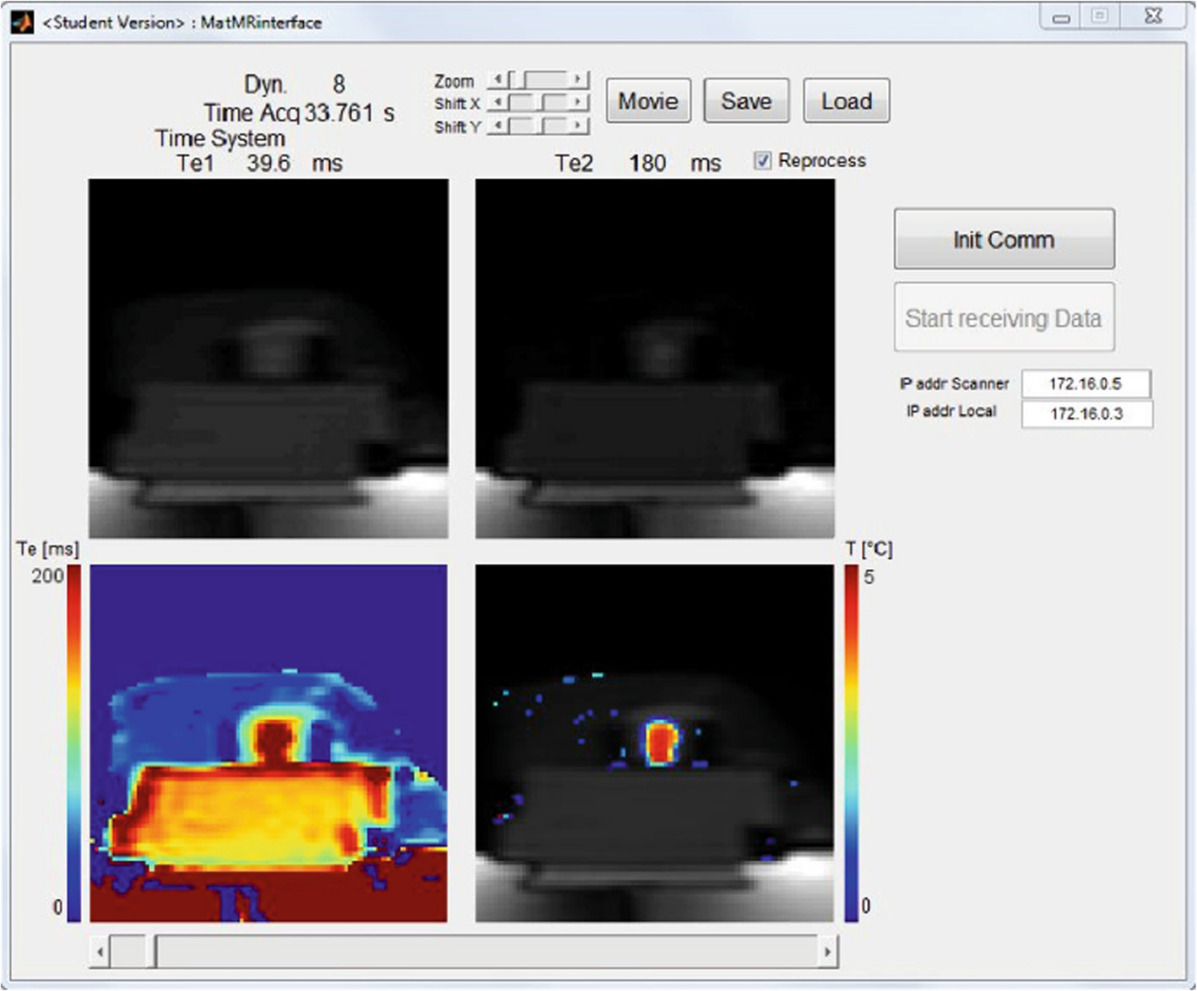
**Graphical user interface for dynamic T2-based thermal mapping in the bone marrow.** Simultaneous echo 1 images, echo 2 images, T2 maps, and T2 change maps (scaled to temperature) are shown.

MatMRI was used to test the hypothesis that changes in bone marrow temperature can be accurately determined by measuring changes in the transverse magnetization decay time (T2) [9] since magnetic relaxation times increase linearly in fat during heating [10,11]. A 2-cm-thick cross section of bovine femur (cut from the diaphysis region) was coupled to the acoustic membrane of the Sonalleve system with degassed water. Calibration of T2-based thermal maps involved heating the marrow in a bovine femur and simultaneously measuring T2 with MRI, and absolute temperature with T-type thermocouples was placed in the bone marrow, cortical bone, and the surrounding soft tissue. The femur received a continuous ultrasound exposure for 60 s with an acoustic power of 50 W. This relatively low power was selected to induce a mild temperature increase of approximately 5°C in the marrow while avoiding irreversible T2 changes to enable correction of T2 and temperature during tissue cooling. Dynamic T2 maps were calculated and displayed in real time during both HIFU exposure and tissue cooling. MR imaging parameters were as follows: FOV = 250 mm, pixel size = 1.5 mm, slice thickness = 5 mm, TE_1_ = 40 ms, TE_2_ = 180 ms, TR = 2.4 s, NEX = 1, and dynamic time = 9.7 s. Figure 3 depicts the user interface for dynamic T2 mapping experiments. Results showed a positive T2 temperature dependence of 20 ms/°C in the bone marrow during the HIFU exposure. The cooling phase showed a temperature dependence of 21 ms/°C, indicating that the measured temperature elevation did not cause irreversible changes in T2 relaxation.

#### Hydrophone measurements with MatHIFU

MatHIFU was used to automate pulsed ultrasound exposures and to perform acoustic pressure measurements. The Sonalleve clinical MR-HIFU system was controlled with MatHIFU to apply ultrasound exposures on a per-element basis and to generate changes in acoustic pressure within the focal region. The pressure was measured using a fiber-optic acoustic hydrophone system (Precision Acoustics, Dorchester, UK). In this application, each element of the transducer was driven one after another using a pulsed driving signal with 40 cycles at 1.2 MHz and with a repetition rate of 40 Hz. An average of 64 signals was calculated using an oscilloscope (MDO4054-3, Tektronix, Beaverton, OR, USA). Using MatHIFU, a protocol was created to automate the ultrasound exposures with the following steps: 

1. Turn off all elements.

2. Turn on only element *n*.

3. Turn on pulsed ultrasound exposure.

4. Turn off ultrasound after 1.8 s of exposure.

5. Remain inactive until all data collected by the oscilloscope are transferred.

Prior to the protocol execution, the oscilloscope was prepared to capture 64 samples of the hydrophone signal over time. Querying the status of the MR-HIFU system was used to determine when the ultrasound exposure was finished, after which the average reading of the 64 acquisitions was transferred through an Ethernet link to the external computer running MatHIFU. Post-transfer, a modification to change the active transducer element was executed and the protocol was restarted. The pseudo-code for the whole acquisition, execution, and modification step of the treatment protocol was as follows:

Do For All Elements In Transducer Prepare Oscilloscope If First Element of Transducer Execute Protocol Else Execute Modification Go to Step 1 of Protocol Wait Until Status of MR-HIFU Indicates End of Ultrasound Exposure Collect Data from Oscilloscope Do Next Transducer Element

**Figure 4:**
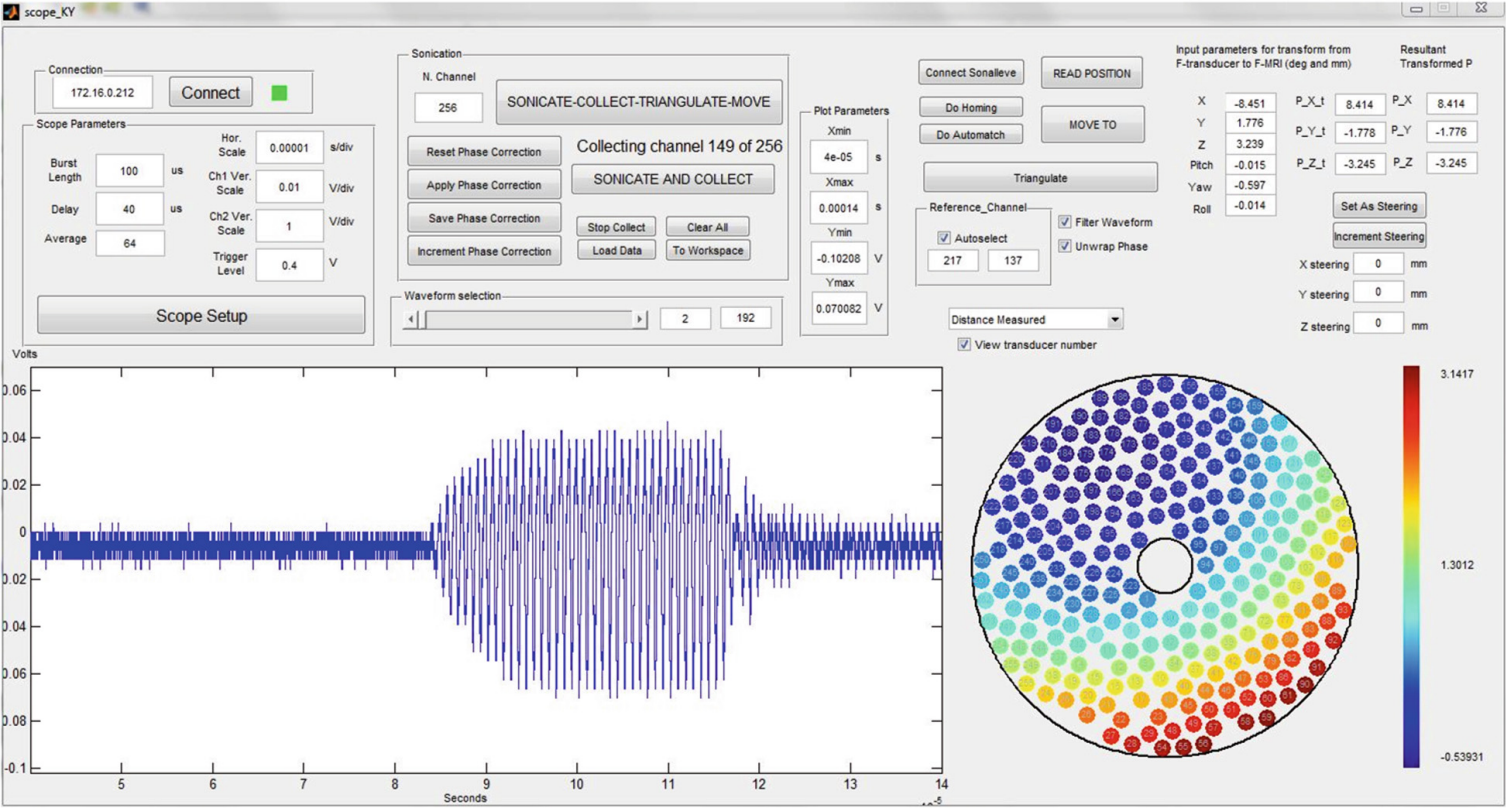
**Graphical user interface used to perform ultrasound exposures and capture the acoustic hydrophone data.** In this example, the acquisition 149 of 256 is being displayed.

Figure 4 shows a screenshot of the graphical user interface that was used to perform and control the ultrasound exposures and hydrophone measurements. The time required to perform an acquisition was 2.3 s per channel and included the overhead for protocol modification, waiting time (1.8 s) to collect 64 acquisitions over time, as well as time to transfer the data. The total time required to capture the measurement data for all 256 transducer elements was 589 s (just under 10 min).

#### The ‘HIFU’ application using MatMRI in conjunction with MatHIFU

**Figure 5:**
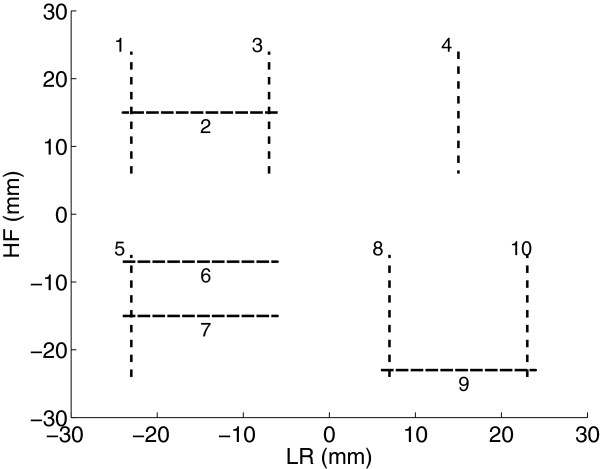
**The heating pattern used to recreate the letters in the word ‘HIFU,’ consisting of straight lines. **The order of the lines is shown using numbers 1 to 10. The transducer was moved first to the center of each letter, and each line was then produced by electronically steering the focal point along the 18-mm-long lines. LR denotes the left-right direction, and HF denotes the head-foot direction.

An application was developed to test and showcase the combined capabilities of MatMRI and MatHIFU. This application was used to produce a heating pattern that would form the letters in the word ‘HIFU’ using straight lines, as shown in Figure 5. A total of ten straight segments were used in the pattern. The heating was performed using a polymer-based tissue-mimicking heating phantom (Philips Healthcare, Vantaa, Finland). Using MatHIFU, a treatment protocol with the following steps was created: 

1. Move the transducer to the center of the letter.

2. Pause for 2 s. This pause is used to acquire a reference image for thermometry.

3. Apply HIFU with an acoustic power of 80 W and electronically steer the focal point in 2-mm steps along the 18-mm-long line. The time between steps (trajectory interval) was set to 25 ms.

4. Turn off the ultrasound after 8 s of exposure.

5. Remain inactive. This allows protocol modifications to be performed.

The coordinates for the transducer position and focal point steering were initially set to produce the first line segment (Figure 5). A cooling time of 60 s was observed after the ultrasound was turned off, and then a protocol modification was created and executed to change the transducer position as well as the steering coordinates. MatMRI was used to collect MR magnitude and phase images that were utilized to calculate temperature change using the PRFS technique. Magnetic drift was monitored in non-heated regions, and a correction was applied [1]. The MR parameters for the thermometry were as follows: FOV = 200 mm, pixel size = 1.1 mm, slice thickness = 7 mm, TE/TR = 20/30 ms, flip angle = 19.5°, acquisition matrix = 180, reconstruction matrix = 224, ETL = 9, NEX = 1, and dynamic time = 0.59 s. Reference images for thermometry were acquired at the beginning of each letter. The protocol duration was 637 s, during which 1,080 images were acquired. The pseudo-code for the image acquisition as well as for the protocol execution and modification steps was:

Do Until All Segments Are Finished If first segment Execute Protocol Else Execute Modification If New Letter Go to Step 1 of Protocol Else Go to Step 3 of Protocol If Transducer Movement Just Finished Collect Magnitude and Phase Image For Reference Else Collect Image Calculate Temperature Change If End of Cooling Do next segment

**Figure 6:**
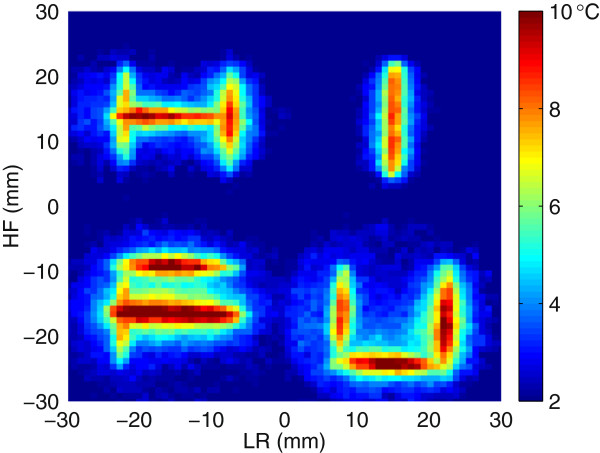
**Maximum observed temperature change projection for the ‘HIFU’ application experiment. **Each of the ten lines in the pattern was produced by electronically steering the focal point along 18-mm-long lines over 8 s. LR denotes the left-right direction, and HF denotes the head-foot direction.

Figure 6 shows the maximum temperature change projection over the protocol execution. The heating pattern reconstructs the word ‘HIFU’ as expected. The peak maximum temperature was 10.4°C over the baseline.

### Discussion

MatMRI and MatHIFU are software tools to perform and facilitate pre-clinical studies. These tools leverage research centers’ multi-million dollar investments in clinical MRI and MR-HIFU hardware, and their greatest strength resides in integrating real-time MRI measurements and controlling the MR-HIFU system with minimal effort. Both MatMRI and MatHIFU toolboxes operate within the MATLAB^®;^ computational software environment, which is well known in many research laboratories. In the present study, four projects were presented to showcase the capabilities of these software tools to simplify and accelerate the development of new MR-HIFU applications. MatMRI was seamlessly integrated with an existing MR-HIFU pre-clinical small animal system. In addition, the development of a new framework to perform T2-based thermometry was facilitated through the use of MatMRI. Furthermore, MatHIFU was used to activate and precisely control the transducer element driving signals of a clinical MR-HIFU system to enable acoustic pressure measurements using a hydrophone. Finally, MatMRI and MatHIFU were combined in a demo application to highlight the capabilities of these tools in pre-clinical thermal therapy applications.

MatMRI substantially simplifies the acquisition and processing of real-time, dynamic MR data, and it may have potential beyond thermal therapy applications. As indicated by the presented examples, applications requiring real-time processing of MR data, such as MR thermometry or functional MRI, may benefit from this software tool. On the other hand, MatHIFU facilitates the exploration of new therapeutic applications using the Philips Sonalleve clinical MR-HIFU system. MatHIFU opens up new opportunities for rapidly adapting this system for application-specific needs in pre-clinical research. MR-HIFU thermal ablation, HIFU-mediated mild hyperthermia and drug delivery, as well as applications utilizing HIFU-induced mechanical effects may benefit from the use of these tools in pre-clinical work.

Both MatMRI and MatHIFU are intended to be freely available as open-source projects to other research groups under the coordination of Philips Healthcare. These tools are meant to facilitate pre-clinical MR-HIFU research and aid collaboration between research teams. The first collaboration under this model initiated by TBRRI took place with the Hospital for Sick Children. The exchange of ideas has been mutually beneficial for both teams, and we aim to expand the community of users to drive the future development of these tools.

## Conclusions

In this study, we presented two software tools to facilitate the research and development of pre-clinical MRI and MR-HIFU applications. MatMRI allows dynamic acquisition and processing of real-time MR data, and MatHIFU allows adaptable and robust control of the Philips Sonalleve clinical MR-HIFU system. Four applications were presented to showcase the capabilities of these tools. MatMRI and MatHIFU may simplify as well as accelerate the development process of new MR-HIFU applications and are meant to foster collaboration between research teams. These tools are intended to be freely available to other research groups under the coordination of Philips Healthcare.

## Methods

All experiments with MatMRI were performed using a Philips Achieva 3.0T TX scanner. MatHIFU experiments were done using the Philips Sonalleve MR-HIFU system.

### MatMRI

MatMRI uses the eXTernal Control (XTC) protocol [12] that was developed to provide low-latency access to MR data as well as to control an MRI scanner in real time. Among other applications, the XTC protocol is used to provide real-time MR data to the Sonalleve clinical MR-HIFU system. A .Net (Microsoft, Redmond, WA, USA) class was written in the C# programming language (Microsoft, Redmond, WA, USA) to encapsulate all the low-level communication protocols. Furthermore, a class in the MATLAB ^*®*^ programming language was written to expose high-level functions to the end user. These functions are utilized to acquire MR data and to control the scanner.

**Figure 7:**
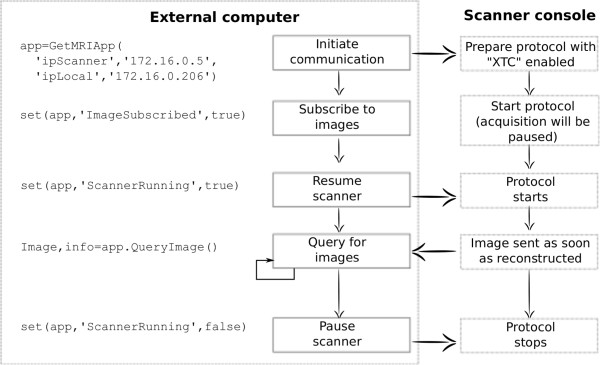
**A flow chart depicting the basic operation of MatMRI. **The actions to be performed on the scanner console as well as on the external computer are shown, including the associated MatMRI instructions.

**Figure 8:**
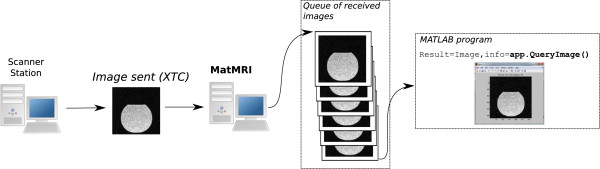
**A schematic describing the process of image acquisition using MatMRI. **Images are sent through XTC as soon they are reconstructed, and MatMRI places them in a queue. The user code in MATLAB ^*®*^ pulls the ‘oldest’ image from the queue and processes it. In practice, assuming that the user code is set to continuously query for new images, the processing is done in real time, and the queue only contains the most recent image.

MatMRI communicates with the MRI scanner through a 1-Gbit/s Ethernet link. Multi-threading capabilities were added to maximize real-time processing speed. An example in which MatMRI is used to acquire individual slices is shown in Figure 7. In this example, image acquisition is initiated on the MRI console and remains paused until the external computer sends an indication to continue. The end-user program subscribes to the MR images and resumes scanning. As shown in Figure 8, the scanner console sends the images, and MatMRI collects them in the background. The end-user program queries for any new image and then processes it. The user program may stop scanning as required. This scenario allows the development of applications that utilize MatMRI for dynamic image acquisition and MATLAB ^*®*^ code for real-time image processing. An example of such application is the calculation of temperature change from temperature-sensitive MR images.

#### Functionality

##### Data acquisition

Available MatMRI functionality includes the acquisition of individual imaging slices as well as volumetric data. The acquisition of individual slices is the preferred choice for real-time imaging because the images are transferred to MatMRI as soon as they are reconstructed. Volumetric acquisition is preferred when acquiring large data sets (multiple 2D slices) in a single dynamic step. The volumetric data are ordered by type, stack, and slice number. Multiple users can subscribe to the data simultaneously. Many different types of images (T1-weighted, T2-weighted, B1 map, etc.), number of slices, stacks, and dynamics can be transferred without any data size limitation related to the active memory of the MR console.

##### Pause/resume image acquisition

MatMRI allows the end user to pause and resume the MR image acquisition. This is useful in applications that will acquire hundreds or thousands of dynamic images and in which it is desirable that the user code controls the acquisition timing.

##### Change imaging stack positioning

The position of each imaging stack as well as its stack orientation are user-redefinable in real time. This function is important for dynamic adjustments of the imaging planes based on user code decisions.

### MatHIFU

**Figure 9:**
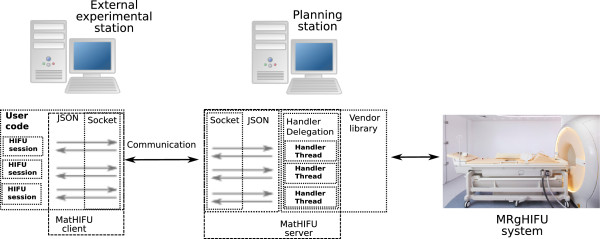
**A schematic illustrating the design of the MatHIFU software tool. **MatHIFU operates as a client-server model where the user establishes sessions that communicate with the server. Each session is handled by its own thread at the server component. The protocol commands are packed as JSON messages, and the communication is realized through TCP sockets. The server processes the commands and performs the required actions using the proprietary library for the MR-HIFU system.

MatHIFU operates as a client-server system (Figure 9) where the server component is executed in a workstation that is part of the standard Sonalleve clinical MR-HIFU system.

The client component is a MATLAB ^*®*^ class that exposes the functions required to prepare custom-made treatment protocols to the user. A treatment protocol is a series of commands, a mini-program in itself, where the parameters, such as ultrasound exposure and transducer positioning, are specified. The client and the server communicate with each other using transmission control protocol (TCP) sockets. JavaScript Object Notation (JSON) is used to encapsulate the communication messages.

**Figure 10:**
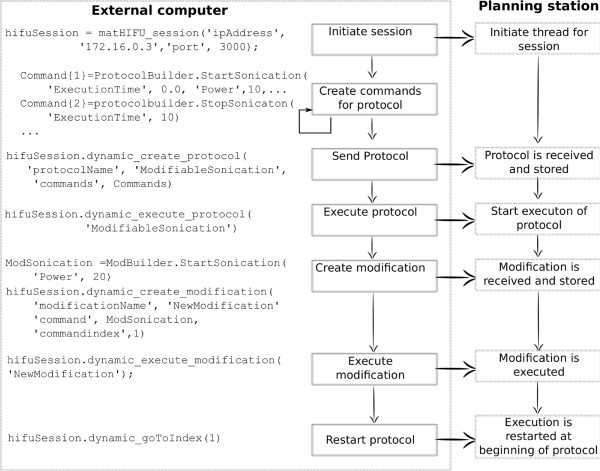
**A flow chart presenting the basic operation of MatHIFU. **It includes the instructions for treatment protocol creation, execution, and real-time modification. In this example, the acoustic power for a continuous ultrasound exposure is adjusted from 10 to 20 W.

The types of commands that can be specified in a treatment protocol include, among others, the position of the transducer, the start and stop of ultrasound delivery, and low-level R&D commands. MatHIFU allows creating, executing, and modifying protocols, each with any number of commands as required by the user. Only one protocol can be executed at a time. It is possible, however, to prepare any number of protocols in advance and execute them one at a time according to application-specific needs. A very important feature of MatHIFU is the modification of a protocol execution in real time. Figure 10 illustrates an example to prepare, execute, and modify a treatment protocol.

#### Functionality

##### Creation of treatment protocols

Each treatment protocol has it its own user-specified identifier such as ‘Protocol_1,’ ‘Heating_Region,’ etc.

The protocols are created with the helper class HIFU_ProtocolBuilder, which contains functions to build the following commands: 

• *Start of continuous ultrasound exposure.* This command initiates a continuous ultrasound exposure and allows, for example, the acoustic power, ultrasound frequency, focal point trajectory coordinates, and trajectory interval to be specified as command attributes. The trajectory can be defined as a series of points where the time at each point (trajectory interval) is specified.

• *Start of pulsed ultrasound exposure.* This command initiates an ultrasound exposure with controllable duty cycle, ultrasound frequency, and trajectory coordinates. It also allows synchronizing each pulse with an external trigger if necessary.

• *Movement of transducer.* This command changes the current position of the transducer with 5 degrees of freedom for 3D Cartesian positioning and two rotation angles over the left-right and head-foot directions.

• *Pausing.* This type of command allows defining ‘non-operational’ states for the MR-HIFU system or waiting for the completion of a previous command. This command facilitates the protocol step timing.

• *R&D command.* This is a very powerful command that allows the user to control the 256-element transducer at a low level. The command allows, among other functions, the selection of active/inactive elements and the application of specific phase and/or amplitude to one or more active elements.

Each of these commands has an index that determines the order of execution. In addition, the commands feature a time index that precisely establishes the time point for command execution.

##### Modification of protocols

Modification commands are similar to the protocol commands. The modifications also have their own identifier (such as ‘Modification_1,’ ‘UpdateExposure,’ etc.) and are created with the helper class HIFU_ModificationBuilder, which contains the functions to build modifications. The most notable difference with the protocol commands is that a modification command specifies the position in the original protocol that will be modified as well as the parameter(s) that is/are to be adjusted.

##### Querying of status

The MR-HIFU system includes several status indicators that can be used in decision making. The indicators that can be queried include (but are not limited to) the following: 

• Current position of the transducer

• Current total forward and reflected acoustic power

• Status of ultrasound exposure (on/off)

• Status of transducer movement (moving/idle)

• Current time index of execution

The status indicators can be used to synchronize events with other software or hardware components. Examples include data acquisitions using oscilloscopes, temperature sensors, and any other external device.

### Contribution of Philips Healthcare

Under a research agreement between Philips Healthcare and the Thunder Bay Regional Research Institute, Philips Healthcare provided libraries, documentation, and example code for the use of the XTC protocol. Philips Healthcare also provided specialized training and example code for the customization of the control of the Sonalleve system.

### Access to toolboxes

As indicated in the ‘Discussion’ subsection, both MatMRI and MatHIFU are aimed to be freely available to researchers in coordination with Philips Healthcare. The tools are hosted in a private repository in Github (http://www.github.com). To gain access to the repository, please contact Samuel Pichardo (http://www.spichard@lakeheadu.ca), with copy to Charles Mougenot (http://www.charles.mougenot@philips.com) and Ari Partanen (http://www.ari.partanen@philips.com). You can also get in contact with Samuel Pichardo through his personal web page (http://www.proteus-mrighifu.net).

## Competing interests

Benjamin Zaporzan, Adam C Waspe, Thomas Looi, and Samuel Pichardo declare that they have no competing interests. Charles Mougenot and Ari Partanen declare that they are employees of Philips Healthcare.

## Authors’ contributions

BZ was the main developer of MatHIFU and participated in the experiments for the applications of the ‘HIFU’ pattern and MR-based thermometry with the small animal MR-HIFU system. ACW, TL, and CM performed the experiments for the applications of heating of bone marrow and hydrophone measurements. CM also provided the basic libraries for data communication with the MRI scanner. AP provided the basic code for the foundation of MatHIFU and performed the editorial process of the manuscript. SP drafted the manuscript, was the main developer of MatMRI, and performed the experiments for the applications ‘HIFU’ and MR-based thermometry with the small animal MR-HIFU system. All authors read and approved the final manuscript.

## References

[B1] Rieke V Butts Pauly K MR thermometry J Magn Reson Imaging 2008 27 2 376 90 10.1002/jmri.21265 18219673 PMC2780364

[B2] Voogt MJ Trillaud H Kim YS Mali WP Barkhausen J Bartels LW Deckers R Frulio N Rhim H Lim HK Eckey T Nieminen HJ Mougenot C Keserci B Soini J Vaara T Köhler MO Sokka S van den Bosch MA Volumetric feedback ablation of uterine fibroids using magnetic resonance-guided high intensity focused ultrasound therapy Eur Radiol 2012 22 2 411 7 10.1007/s00330-011-2262-8 21901565 PMC3249029

[B3] Quesson B Merle M Köhler M Mougenot C Roujol S de Senneville B Moonen C A method for MRI guidance of intercostal high intensity focused ultrasound ablation in the liver Med Phys 2010 37 2533 10.1118/1.3413996 20632565

[B4] Hynynen K MRI-guided focused ultrasound treatments Ultrasonics 2010 50 2 221 9 10.1016/j.ultras.2009.08.015 19818981

[B5] Ranjan A Jacobs GC Woods DL Negussie AH Partanen A Yarmolenko PS Gacchina CE Sharma KV Frenkel V Wood BJ Dreher MR Image-guided drug delivery with magnetic resonance guided high intensity focused ultrasound and temperature sensitive liposomes in a rabbit Vx2 tumor model J Control Release 2012 158 3 487 94 10.1016/j.jconrel.2011.12.011 22210162 PMC3319290

[B6] Hijnen N Heijman E Köhler M Ylihautala M Ehnholm G Simonetti A Grüll H Tumour hyperthermia and ablation in rats using a clinical MR-HIFU system equipped with a dedicated small animal set-up Int J Hyperthermia 2012 28 2 141 55 10.3109/02656736.2011.648137 22335228

[B7] Partanen A Yarmolenko PS Viitala A Appanaboyina S Haemmerich D Ranjan A Jacobs G Woods D Enholm J Wood BJ Dreher MR Mild hyperthermia with magnetic resonance-guided high-intensity focused ultrasound for applications in drug delivery Int J Hyperthermia 2012 28 4 320 36 10.3109/02656736.2012.680173 22621734 PMC7641882

[B8] Rieck B Curiel L Mougenot C Zhang K Pichardo S Treatment of localized abscesses induced by methicillin-resistant Staphylococcus aureus (MRSA) using MRgFUS: first in vivo results AIP Conference Proceedings - 12th International Symposium on Therapeutic Ultrasound June 10–13 2012: Heidelberg, Germany. Volume 1503 2012 Melville: AIP 173 178

[B9] Waspe A Looi T Mougenot C Amarai J Temple M Sivaloganathan S Drake J Dynamic T2-mapping during magnetic resonance guided hifu ablation of bone marrow AIP Conference Proceedings - 12th International Symposium on Therapeutic Ultrasound June 10–13 2012: Heidelberg, Germany. Volume 1503 2012 Melville: AIP 222 226

[B10] Hey S de Smet M Stehning C Grull H Keupp J Moonen CT Ries M Simultaneous T1 measurements and proton resonance frequency shift based thermometry using variable flip angles Magn Reson Med 2012 67 2 457 63 10.1002/mrm.22987 22052363

[B11] Todd N Diakite M Payne A Parker DL Hybrid proton resonance frequency/T1 technique for simultaneous temperature monitoring in adipose and aqueous tissues Magn Reson Med 2013 69 62 70 10.1002/mrm.24228 22392856 PMC3371281

[B12] Smink J Häkkinen M Holthuizen R Krueger S Ries M Berber Y Moonen C Köhler M Vahala E eXTernal Control (XTC): a flexible, real-time, low-latency, bi-directional scanner interface Proceedings of the 19th Annual Meeting of ISMRM; May 7–13 Montréal, Québec, Canada 2011 Berkeley: ISMRM 1755 1755

